# Comparative analysis of *de novo* transcriptome assembly

**DOI:** 10.1007/s11427-013-4444-x

**Published:** 2013-02-08

**Authors:** Kaitlin CLARKE, Yi YANG, Ronald MARSH, LingLin XIE, Ke K. ZHANG

**Affiliations:** 1 Bioinformatics Core, Department of Pathology, University of North Dakota, Grand Forks, ND 58202, USA; 2 Department of Computer Science, University of North Dakota, Grand Forks, ND 58202, USA; 3 Department of Biochemistry and Molecular Biology, University of North Dakota, Grand Forks, ND 58202, USA

**Keywords:** transcriptome assembly, next-generation sequencing, RNA-Seq, De Bruijn graph, overlap graph

## Abstract

The fast development of next-generation sequencing technology presents a major computational challenge for data processing and analysis. A fast algorithm, de Bruijn graph has been successfully used for genome DNA *de novo* assembly; nevertheless, its performance for transcriptome assembly is unclear. In this study, we used both simulated and real RNA-Seq data, from either artificial RNA templates or human transcripts, to evaluate five *de novo* assemblers, ABySS, Mira, Trinity, Velvet and Oases. Of these assemblers, ABySS, Trinity, Velvet and Oases are all based on de Bruijn graph, and Mira uses an overlap graph algorithm. Various numbers of RNA short reads were selected from the External RNA Control Consortium (ERCC) data and human chromosome 22. A number of statistics were then calculated for the resulting contigs from each assembler. Each experiment was repeated multiple times to obtain the mean statistics and standard error estimate. Trinity had relative good performance for both ERCC and human data, but it may not consistently generate full length transcripts. ABySS was the fastest method but its assembly quality was low. Mira gave a good rate for mapping its contigs onto human chromosome 22, but its computational speed is not satisfactory. Our results suggest that transcript assembly remains a challenge problem for bioinformatics society. Therefore, a novel assembler is in need for assembling transcriptome data generated by next generation sequencing technique.

A next-generation sequencing technology, RNA-Seq, has rapidly become a major tool for quantifying transcriptome for various organisms [1,2]. If research is conducted for human or model species whose reference genome is known, the transcriptome can be constructed by mapping the short reads of RNA-Seq onto the reference genome. Nevertheless, the genome sequences of most non-model species are not available. Therefore, transcriptome construction and quantification have to rely on transcriptome assembly that is technically difficult. The development of assemblers for RNA-Seq faces two major technical challenges. First, the enormous number of short reads poses high requirements for computational speed and memory efficiency. For mammals and plants, the number of short reads typically ranges from 20 to 100 million for one sample. Pairwise alignments of all short reads are computationally costly; the assembly usually has terabytes of input and intermediate data, posing a big challenge for memory loading and data traffic. Second, a gene of eukaryotic cell often encodes multiple transcripts that share exons between each other. Different genes may also have consensus sequences. These shared sequences of RNA fragments may result in incorrect concatenation of short reads.

Currently there are two major types of assembly algorithms for genome sequences: overlap graph and de Bruijin graph [3]. Overlap graph is based on pairwise alignment between short reads [4]. In the graph, each node represents one short read, and an edge between two nodes indicates the two short reads have overlapping sequences. After some steps of simplifying the overlap graph by removing the transitive nodes and edges, a chain of nodes elicits the sequence of a contig or a transcript. Most overlap graph assemblers were developed in the late 1990s and early 2000s for shotgun genome sequencing. Mira [5], Phusion [6] and Newbler [7] are a few of the widely used overlap graph-based assembly programs. However, the step of making pairwise alignments makes it computational forbidden to use overlap graph methods for assembling the huge number of short reads generated by the next-generation sequencing techniques. This stimulated researchers to develop new assembly programs based on de Bruijn graph [8], a fast graph algorithm. In de Bruijn graph, short reads were broken down into short DNA sequences of length *k*, referred as *k*-mers. *k*-mers are then used to form the graph. Thus, the time complexity for construction of de Bruijn graph is linear. Three prominent de Bruijn assemblers are ABySS [9], Velvet [10] and Trinity [11]. Another widely used transcriptome assembler, Oases [12], is built on top of Velvet by taking into account alternative splicing. A major drawback of de Bruijn graph algorithm is the loss of short read information when splitting reads into shorter *k*-mers. When two genes have a shared sequence of a length greater than *k*, de Bruijn graph would incorrectly connect the reads from these two genes. Therefore, transcriptome assembly remains a challenge for bioinformatics community to balance the computing efficiency and assembly accuracy.

In this study, we evaluated and compared the performance of five commonly used assemblers, Mira, ABySS, Velvet, Oases and Trinity for next-generation sequencing short reads generated from simulation, spike-in RNAs and human brain tissue. The assembly performance is evaluated by a set of statistics, including the number of contigs, N50 length, the rate of short reads mapping onto contigs and the rate of contigs mapping onto transcripts. To investigate the effect of the sequence depth on assembly, we selected varying number of short reads for the experiments. Our work demonstrated the challenges and the need of developing new algorithms for transcriptome assembly.

## 1 Materials and methods

### 1.1 *De novo* assembly tools

Five assemblers, Mira (v3.4.0.1), ABySS (v1.3.3), Velvet (v1.2.03), Oases (0.2.06) and Trinity (r2012-06-08) were selected for this study. These tools include two types of assembly algorithms. Mira is based on overlap graph algorithm, whereas the other assemblers are all based on de Bruijn graph algorithm.

### 1.2 Data simulation

A 5000000 base pair (bp) region in human chromosome 22 between genomic positions 35000000 and 40000000 was used for generating simulated short reads. The exon and transcript sequences in this genomic region were obtained from UCSC known genes dataset [13]. Short reads were generated from the transcript sequences at a random start position. Each short read sequence has equal chance to be made from the sense or anti-sense strand. A pre-assigned number (between 50 and 500) of short reads were generated for each transcript and each short read was 100 bp in length. If the sequence length between the start position and the end of the transcript is less than 100 bp, the short read would be removed. Therefore, the resulting number of short reads was often less than the pre-assigned number for a transcript. For each pre-assigned number of short reads, 10 replications were performed.

### 1.3 ERCC data

The RNA-Seq data for the External RNA Control Consortium (ERCC) RNAs [14] were provided to us by Beijing Genomics Institute, Shenzhen, China. The sample contains 92 ERCC RNAs that have various concentrations. Bowtie [15] was used to map the short reads to the 92 RNA templates. Ten RNAs with the highest concentrations (the highest number of short reads mapped) were selected resulting a total of 1.5 billion short reads. A random set of short reads were selected for assembly. The numbers of selected reads were 20000, 50000, 100000 and 200000 respectively. Each experiment was repeated three times.

### 1.4 Human brain RNA-Seq data

The RNA-Seq data for human brain tissue was collected by Illumina [16]. We selected all short reads that were mapped onto chromosome 22 using Bowtie. For each test of comparing assemblers, a random set of short reads mapped to chromosome 22 were selected. The numbers of selected short reads were between 200000 and 1500000. The set of short reads was also mapped onto chromosome 22 and their transcript template sequences were inferred from Ensembl Human Genome release 69 using Tophat and Cufflink [17,18]. The assembled contig sequences were then compared with the transcript template sequences. For each number of short reads, three experiments were conducted for replications.

### 1.5 Statistical analysis and programming

The five programs assembled the short reads of each RNA-Seq dataset into contigs. The performances of the five assemblies were compared using a set of statistics. For each test data, the exact number of transcript templates was known. Thus, the number of contigs in each assembly was compared with the expected number of transcripts. N50 is a commonly used statistic for assembly evaluation. It is a weighted median of the lengths of contigs [19]. For genome sequencing, a larger N50 value indicates a better performance for the assembly. For transcriptome sequencing, though N50 is not as important, it is still a valuable measurement in our tests because the median transcript length is known. The rate of short reads mapping onto contigs indicates the amount of input information preserved by an assembly. The rate of contigs mapping onto transcripts indicates the accuracy of assembled contigs.

## 2 Results

### 2.1 The de Bruijn graph algorithm generates mis-assembled contigs

Although de Bruijn graph is computationally efficient, this algorithm potentially mis-link short reads because it is based on *k*-mers. If two transcripts that are encoded by distinct genes share a same *k*-mer, they will be mistakenly aligned and connected. The error rate of de Bruijn graph can be estimated by counting the number of pairs of transcripts that have at least one shared k-mers. This error rate may differ between species. In this study, we used human transcript data to estimate the error rate generated by de Bruijn graph.

**Figure 1:**
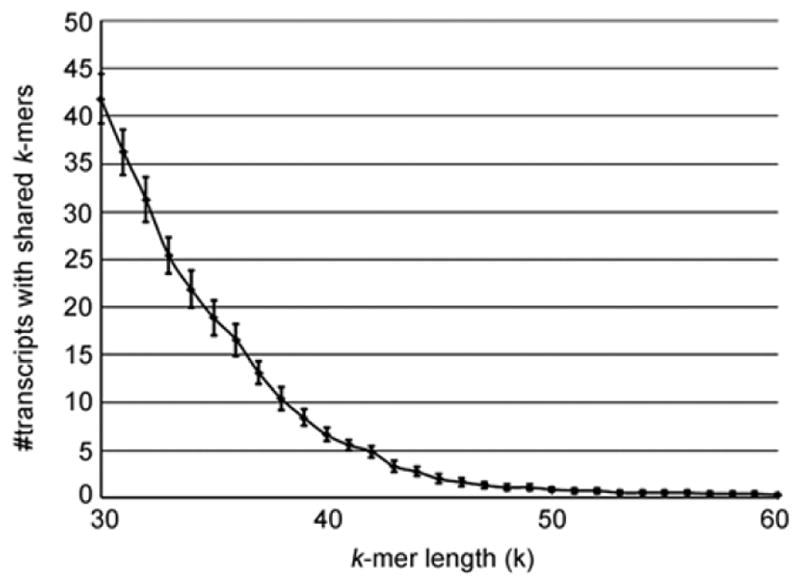
The numbers of transcript pairs that have shared *k*-mers. The total number of transcript pairs is 10000. The errors bar show the standard errors.

We obtained 77600 human transcript sequences using UCSC Known Gene dataset. In order to find the chance that a pair of transcripts has a shared k-mer, we randomly selected 10000 pairs of transcripts and count the number of pairs that have shared *k*-mers. The lengths of *k*-mers ranged from 30 to 60 base pairs. The test was performed for nine times and the results are shown in Figure 1. For *k*=30, an average of 42 transcript pairs were found to have shared *k*-mers. This number dropped below 5 when the length of *k*-mer was large than 41. Many de Bruijn graph-based assemblers recommend a relative small *k*. For example, the default *k* for Velvet and Oases is 31. Our results suggested that a larger *k*-mer size should be used for assembly when using de Bruijn algorithm.

### 2.2 Simulation study

A eukaryotic gene often consists of numerous exons and introns that encode multiple transcripts. Such complicated gene structure presents a major challenge for eukaryotic transcriptome assembly. In order to simulate the process of eukaryotic transcriptome assembly, we select a small genomic region in human chromosome 22 between genomic positions 35000000 and 40000000 for generating random short reads. This small genomic region allows for fast computation while maintaining necessary complexity in gene structure. There are 187 genes that are located in this region encoding 2528 exons and 337 transcripts. By average, each gene consists of 14 exons and encodes 2 transcripts. The average length of exons is 315 base pair (bp), and the median length is 135. Although the largest exon has 9795 nucleotides, approximately 90% of exons have less than 500 nucleotides. Figure 1 shows the distribution of transcript lengths with a mean length of 2359 bp.

**Figure 2:**
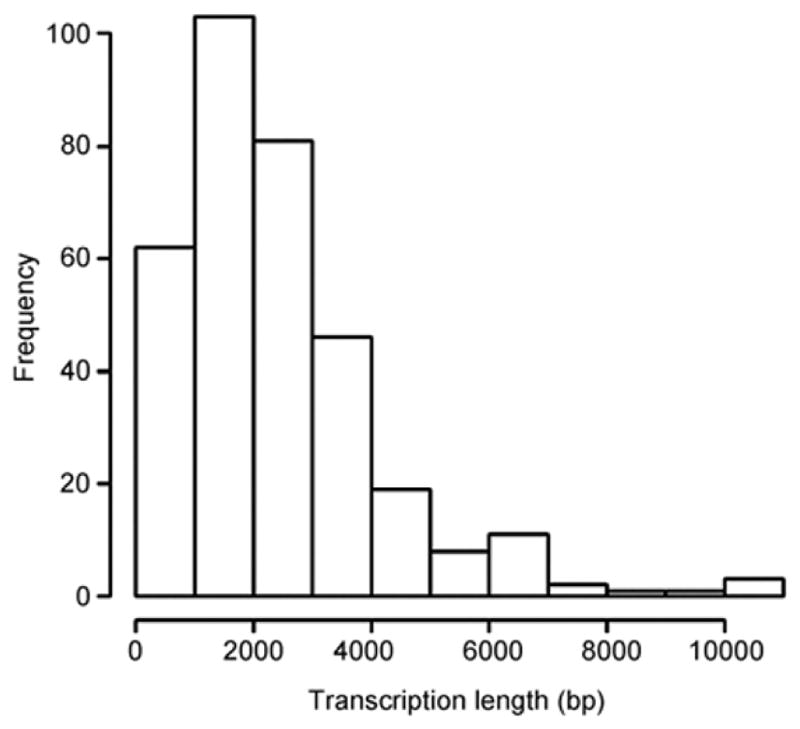
The distribution of transcript lengths. The histogram shows the lengths of 337 transcripts that are encoded in human chromosome 22 between position 35000000 and 40000000.

To simulate the transcriptome sequencing, we randomly generate a number of short reads with 100 bp in length for each transcript. The start position of each short read was randomly selected. If the start position is close to the end of a transcript such that the resulting short read is less than 100 bp, the read would be removed. No sequencing errors were introduced into the simulation. Each read has an equal chance to be synthesized from either the sense strand or the missense strand. The number of short reads per transcript ranged from 50 to 500. Each simulation was repeated 10 times and the average statistics were calculated (Figure 2). The standard errors of all statistics were so small that they were invisible in Figure 2.

Figure 2A shows the number of contigs generated by each assembler. The expected number of contigs is 337 because the short reads were generated from 337 transcripts. ABySS, Velvet and Oases converged to 337 as the number of short reads increases and they (by average) generate 306.5, 427.5 and 367.5, respectively, when there are 500 short reads for each transcript. Trinity stabilized very fast and it consistently generated more than 600 contigs for at least 100 reads per transcript. Mira had the least performance in terms of number of contigs. Its number of contigs almost linearly increased and it was above 1000 when there were at least 300 reads per transcript.

N50 statistic is commonly used in assembly evaluation. It measures the weighted median of the contig lengths. N50 is an important statistic for genome assembly because a larger value indicates that the assembler create less break points in the genome. However, a larger N50 does not necessarily imply better performance for transcriptome assembly be cause transcriptome are fragmented DNA sequences. In the simulation, it is known the average transcript length is 2359. Therefore, we evaluate the performance of assemblers by the distances of their N50 to 2359. Both Oases and Trinity had acceptable performance with N50s slightly greater than 2359 (Figure 2B). All the other assemblers had N50s less than 1500, indicating they generated too many small con-tigs.

Mira and Trinity both had excellent short read mapping rate, approximately 90% of short reads mapped onto the assembled contigs, whereas other assemblers had less optimal performance (Figure 2C). In terms of contig mapping rate, ABySS and Velvet had about 95% contigs that can be mapped to the transcripts (Figure 2D), whereas the rates were approximately 90%, 46% and 8% for Mira, Trinity and Oases, respectively.

### 2.3 ERCC data

**Figure 3:**
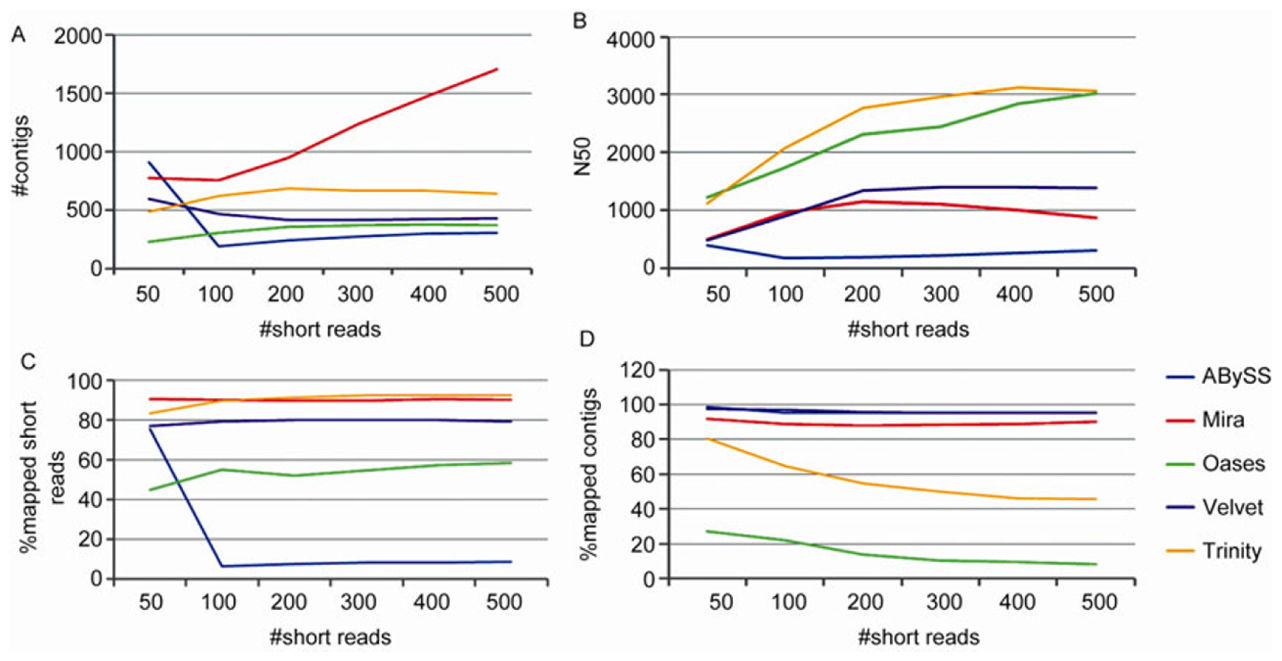
Comparison of assembly statistics for simulated transcriptome data. The *x* axis indicates the number of short reads generated for each transcript. A, The number of contigs from each assembler. B, N50 statistics. C, The percentage of short reads that can be mapped to the contigs. D, The percentage of contigs that can be mapped to the original 337 transcripts.

The External RNA Control Consortium (ERCC) has developed a set of 92 RNAs that have been widely used in quality control for microarray, qPCR and next-generation sequencing for quality control [14,20]. The mean length of ERCC RNAs is 900 bp. A mix of ERCC RNAs was deeply sequenced using Illumina Hiseq platform. The concentrations of the 92 RNAs are highly variable in the ERCC mix. Some RNAs are highly expressed and some other RNAs are rarely expressed. In order to test assembly algorithms, we selected only 10 RNAs that had the highest concentrations in the ERCC mix. A total of 1.5 billion short reads were mapped to these 10 RNAs using Bowtie [15]. For each test, we randomly selected a set of short reads for assembly. The numbers of selected reads were 20000, 50000, 100000 and 200000 respectively. Each experiment was repeated three times. The summary statistics are shown in Figure 3.

In Figure 3A, both Oases and Trinity gave the best performance. For 200000 short reads, Oases generated exactly 10 contigs as expected from the 10 selected RNAs, and Trinity generated 10.7 contigs by average. ABySS had 6.3 contigs. The results for Velvet and Mira were not optimal, both giving hundreds of contigs. Similarly, Oases and Trinity had N50 values that were close to the mean RNA length of 900 bp (Figure 3B). The other three assemblers gave significantly lower N50 values. Trinity had almost perfect results (close to 100%) for short reads mapping rates and con-tig mapping rates (Figure 3C and D). Mira also had higher mapping rates than the other assemblers and its rates increased as the number of short reads increased. The mapping reads of the other three assemblers were below 60% for 200000 short reads.

### 2.4 Human Chromosome 22 data

The five assemblers were compared using human brain RNA-Seq data. In order to facilitate the computation, the assembly was only performed on the short reads that mapped to human chromosome 22. We collected 7.8 million short reads in total for chromosome 22. For each test, we randomly selected a number of short reads for assembly. The numbers of short reads were between 200000 and 1500000. Each test was repeated three times. The transcripts that can be formed by the selected short reads were also identified using Tophat and Cufflinks on Ensembl Human Genome release 69 [17,18]. The assembly was evaluated by comparing contig sequences with the transcript sequences that were from the known human genome sequence.

**Figure 4:**
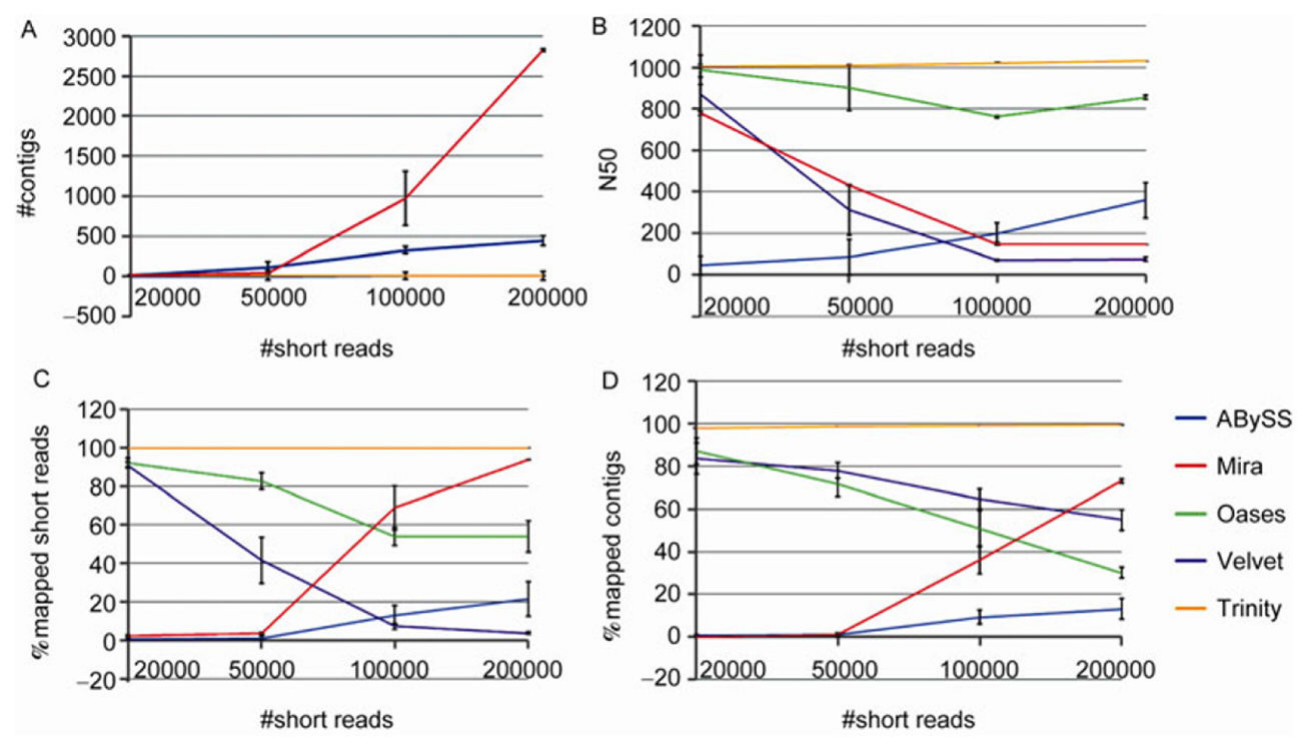
Comparison of assembly statistics for ERCC data. The *x* axis indicates the total number of selected short reads for assembly. A, The number of contigs from each assembler. B, N50 statistics. C, The percentage of short reads that can be mapped to the contigs. D, The percentage of contigs that can be mapped to the 10 RNA templates.

In Figure 4A, the black line shows the number of transcripts identified by Cufflinks. Oases generated the closest number of contigs. Trinity also had a close number of con-tigs. All the other three assemblers had too many contigs. In Figure 4B, Oases had the largest N50. Mira, Trinity and Velvet all had the best rates for short reads mapping onto transcripts (Figure 4C). Trinity, Mira and Oases had the best rates for contigs mapping to transcripts, whereas ABySS had the low mapping rate for short reads and contigs (Figure 4C and D).

## 3 Discussion

Transcriptome sequencing is becoming a dominant technique to quantify the global gene expression driven by the rapidly dropping cost of next generation sequencing. For species without reference genomes, the first step of analysis is to assembly the raw short reads, a computationally complicated process. Most of assembly algorithms were originally developed for genome sequence, nevertheless the assembly of eukaryotic transcriptome is substantially more difficult because a single gene often encodes multiple transcripts by alternative splicing of a limited number of exons. It remains a question whether current assembly tools can meet the rapidly increasing demand of transcriptome sequencing [1,2]. This motivated us to perform an extensive comparison and evaluation for some of the most widely-used assembly programs using both simulated and real transcriptome data.

Five assembly tools were tested for three RNA-Seq data types: simulated data, ERCC data and human brain tissue data at various sequencing depths. The RNA-Seq simulation used human transcript sequences while assuming ideal conditions with equal numbers of short reads per transcript and without sequencing errors. The simulation results reflected the effects of complicated gene structures of eukaryotic cells on assembly. ERCC data consists of a number of RNA templates. Because there is no significant overlap between RNA template sequences, ERCC RNA-Seq is a simplified transcriptome that contains alternative splicing isoforms and complicated gene structures. In order to test for eukaryotic transcriptome, we selected a subset of short reads from human brain RNA-Seq experiment. All the short reads were mapped to chromosome 22. We chose different number of short reads ranging from 200 K to 1.5 M. Approximately 800 K short reads in chromosome 22 were equivalent to 100 M short reads for the whole human genome. Currently most human transcriptome studies employ between 20 and 100 M short reads per sample. Thus, our tests provided equivalent sequence depths to real studies.

Our testing results showed that the assembly tools had varying levels of performances for different data types and sequence depths. Mira gave good mapping rates for short reads and contigs, which was expected because it used a slower but more accurate algorithm, overlap graph. However, Mira’s assembly contained far more contigs than what is expected, and most of contigs were short. This indicated that Mira was not suitable for transcriptome assembly because of the complicated gene structures in eukaryotes. ABySS had low mapping rates for all three data types. Trinity had excellent mapping rate for short reads in simulated data, but the contig mapping rate was low. Trinity had good performance in mapping short reads to contigs and mapping contigs to transcripts for both ERCC and human chromosome 22 data. However, its N50 was not the best, implying Trinity could always generate the full length transcripts. As a revised version of Velvet for transcriptome assembly, Oases had better number of contigs and N50 than Velvet. Oases had a better short read mapping rates than Velvet in most of times. However, the contig mapping rate for Oases was not as good as Velvet for all of the tests.

In summary, no assembler had consistent good performance in all the statistics. For transcriptome assembly of prokaryotic cells that have simple gene structure, Trinity would be recommended. For eukaryotic genome, both Oases and Trinity gave acceptable performance. The development of transcriptome assembler remains a challenge for future genome studies.

## Figures and Tables

**Figure 5:**
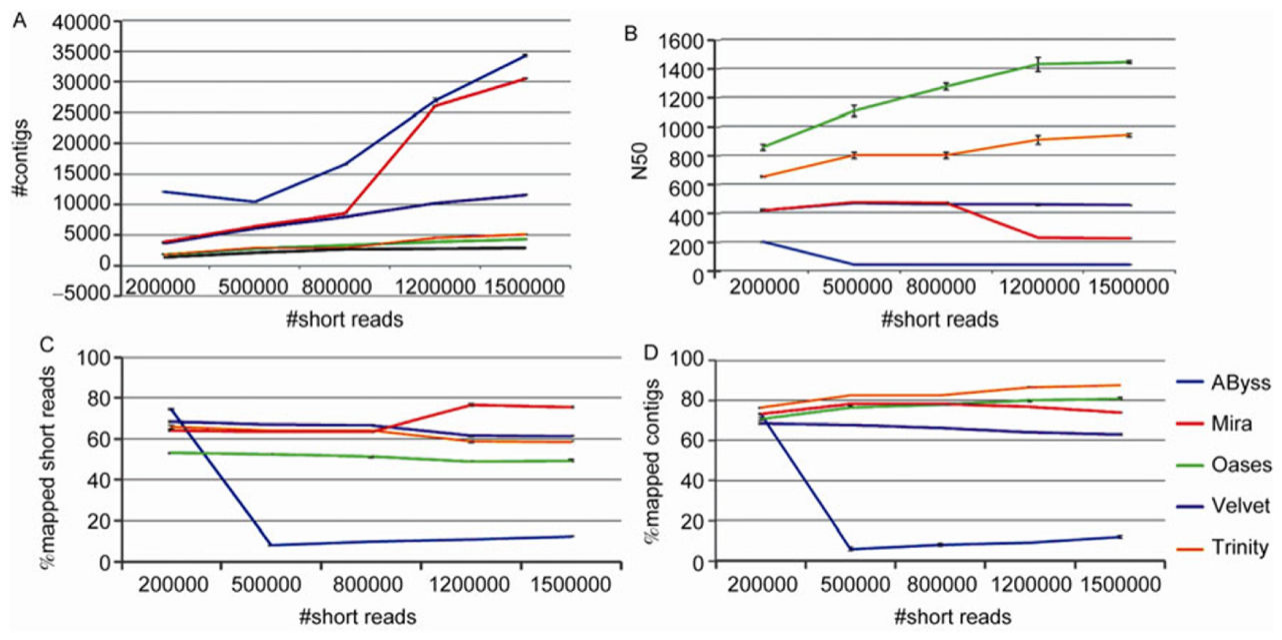
Comparison of assembly statistics for human brain data. The *x* axis indicates the total number of selected short reads that were mapped to chromosome 22. A, The number of contigs from each assembler. B, N50 statistics. C, The percentage of short reads that can be mapped to the contigs. D, The percentage of contigs that can be mapped to the transcripts identified by Cufflinks.
